# A Review on Catalytic Nanomaterials for Volatile Organic Compounds VOC Removal and Their Applications for Healthy Buildings

**DOI:** 10.3390/nano9060910

**Published:** 2019-06-23

**Authors:** Kwok Wei Shah, Wenxin Li

**Affiliations:** Department of Building, School of Design and Environment, National University of Singapore, 4 Architecture Drive, Singapore 117566, Singapore; bdglw@nus.edu.sg

**Keywords:** nanomaterials, VOCs removal, photocatalysis, thermal oxidation, catalytic oxidation, healthy buildings, green application, photocatalytic reactor

## Abstract

In order to improve the indoor air quality, volatile organic compounds (VOCs) can be removed via an efficient approach by using catalysts. This review proposed a comprehensive summary of various nanomaterials for thermal/photo-catalytic removal of VOCs. These representative materials are mainly categorized as carbon-based and metallic oxides materials, and their morphologies, synthesis techniques, and performances have been explained in detail. To improve the indoor and outdoor air quality, the catalytic nanomaterials can be utilized for emerging building applications such as VOC-reduction coatings, paints, air filters, and construction materials. Due to the characteristics of low cost, non-toxic and high chemical stability, metallic oxides such as TiO_2_ and ZnO have been widely investigated for decades and dominate the application market of VOC-removal catalyst in buildings. Since other catalysts also showed brilliant performance and have been theoretically researched, they can be potential candidates for applications in future healthy buildings. This review will contribute to further knowledge and greater potential applications of promising VOC-reducing catalytic nanomaterials on healthier buildings for a better indoor and outdoor environment well-being.

## 1. Introduction

Healthy buildings aim to provide a healthy built environment for occupants inside buildings, and indoor air quality can significantly impact occupants’ health [1]. Since people spend most of their time inside buildings, indoor air quality has become an increasing concern. Indoor air quality can be affected by various factors including toxicological, microbiological, physical systems, indoor, and outdoor ventilations [2]. The advances in construction technology have produced numerous applications of synthetic building materials, but have also brought some adverse effects on the indoor environment, including the major pollution from volatile organic compounds (VOCs). VOCs are defined as having a boiling point that ranges between 50 °C and 260 °C [3]. They can contribute to the formation of ozone and fine particulates in the atmosphere [4] and work directly as toxic substances to the environment and human being. The exposure to VOCs can result in both acute and chronic health effects including respiratory diseases, impaired neurobehavioral function, and sick building syndrome, etc. [2].

**Figure 1:**
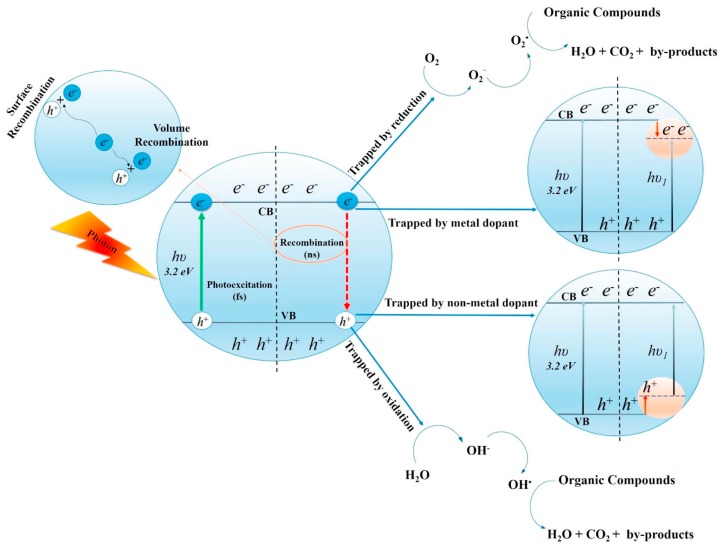
Mechanisms of photocatalytic oxidation for the removal of VOCs [14]. (reproduced from [14], with permission from Elsevier, 2019).

In order to remove the VOCs, many methods have been proposed and can be roughly divided into two main groups according to their mechanisms: adsorption techniques and oxidation techniques [5], or the combination of them [6]. The former one is a conventional method by transferring VOCs from the air to the solid phase via adsorbents, e.g., activated carbon [7], biochar [8] and fibre [9], etc. which faces challenges like saturation and pore blockages. The latter oxidation techniques provide a better approach to cost-effectively remove VOCs, which show higher degradation activity towards polar VOCs (OVOCs > Ahs > AlHs) [10]. Photo and thermal catalytic oxidation are two of the most common oxidation techniques. Thermal oxidation reactions require a high temperature above 600 °C, and the oxidation efficiency grows with the temperature increase. The technique of photocatalytic oxidation commonly uses nano-semiconductor catalysts and ultraviolet (UV) light to convert organic compounds in indoor air into benign and odourless constituents—water vapor (H_2_O) and carbon dioxide (CO_2_) for air purification [5,11,12]. Figure 1 shows the basic principle of photocatalytic oxidation for the removal of VOCs. An electron in an electron-filled valence band (VB) is excited by photoirradiation to a vacant conduction band (CB), leaving a positive hole in the VB. Later, the photogenerated electrons and holes can react with H_2_O and O_2_ molecules to reduce and oxidate VOCs on the surface of a photocatalyst [12,13,14].

Most of the VOCs in indoor air are aromatics, aldehydes, and halocarbons, and they are rich in the established, new and renovated buildings [15]. Measurements indicate that similar exposure level is shared by VOCs in various indoor and building materials, and coverings are the major sources of VOCs [16]. VOCs are common in industry [5] and widely used in construction projects. Since many VOCs will off-gas a significant proportion of their volume in a relatively short time, the VOC concentrations could be much higher than typical ambient levels in newly-constructed or decorated buildings [2]. To reduce the VOC emissions and indoor concentrations inside the buildings, some proposed to bake-out the housing unit with a radiant floor-heating system [17], and a long-enough bake-out could deplete solvents and reduce VOC emissions [18]. While a more efficient method is to adopt photocatalysis such as TiO_2_, these materials can be applied on buildings as a coating or paint, or synthesised with mortar, which will further affect the aesthetics, sanitation and efficiency of the buildings [19,20,21]. Therefore, special attention should be paid to the construction materials.

**Figure 2:**
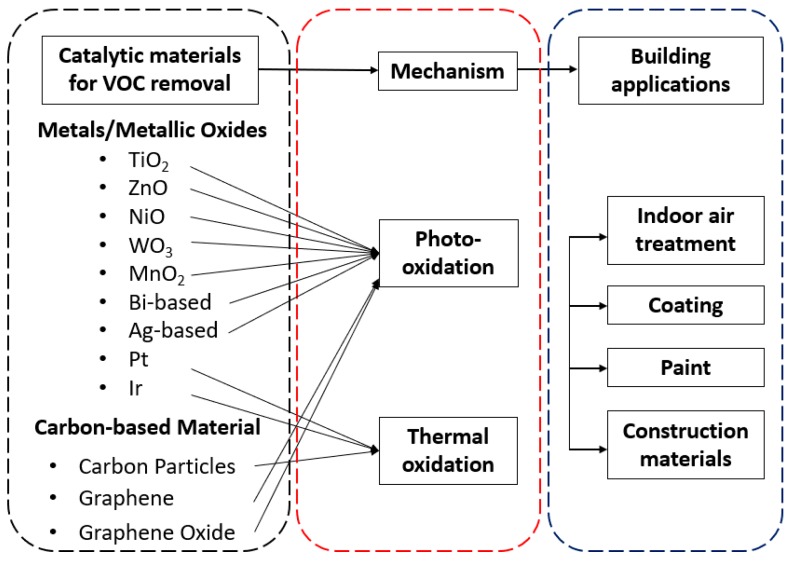
Outline of this review paper.

Since the topic has been investigated for decades, many researchers have summarized the materials for the removal [5,22] and sensing [23,24] of VOCs. Most references have been reviewed based on a specific category of material, such as TiO_2_ [13,25,26], graphene-based materials [27], zinc indium sulphide [28] and silica-nanosphere-based materials [29] etc., or focusing on the catalytic oxidation processes in a specific situation such as low temperature [30], visible light [31,32], or based on a summary from the perspective of various VOCs [5]. However, few have been done from a material perspective together with the consideration of applications on buildings. In this review paper, we do not discuss adsorption-based materials because there are already existing review papers [5,9]. Since the performances of the catalytic oxidations are different for various materials, it is reasonable to categorize by their physiochemical characteristics, which will further affect their applications on buildings. Therefore, this review proposed a novel summary on the materials for catalytic removal of VOCs and their applications on buildings (Figure 2). Morphology, synthesis, performance, and their applications will be explained in detail. This review will further contribute to the applications of photocatalysis materials on buildings.

## 2. Materials

This review will summarize the materials used for catalytic oxidation of VOCs. The commonly used materials and their characteristics have been listed in Table 1. For this study, catalysts used for the oxidation of VOCs can be classified into two major groups: metallic oxide catalysts and carbon-cased materials.

### 2.1. Metallic Oxides

#### 2.1.1. Titanium Dioxide (TiO_2_)

With a wide band-gap energy, durability against photo-corrosion, low toxicity, and low cost, TiO_2_ is regarded as the most efficient and applicable material [59]. In 1972, the photoelectrochemical decomposition of water under irradiation with light on TiO_2_ was found for the first time [60]. Photocatalysis performances of TiO_2_ and its derivatives were studied over decades.

The photocatalyst performance can be affected by the structure and morphology [33] and treatments [34] of the TiO_2_ particles. Maira et al. [33] investigated the effects of different synthesis parameters on the size and morphology of the TiO_2_ particles, and the trichloroethylene degradation over TiO_2_ catalyst exhibits a maximum at a primary particle size of 7 nm. They [34] further compared the treatments applied to an amorphous TiO_2_ precursor for obtaining nanosized TiO_2_ particles. Compared to the anatase TiO_2_ treated with the thermal method, the one with the hydrothermal method can improve the photo activity and showed a higher number of hydrogen-bonded hydroxyl groups that are more stable under RT outgassing and a stronger adsorption ability on Benzaldehyde.

**Figure 3:**
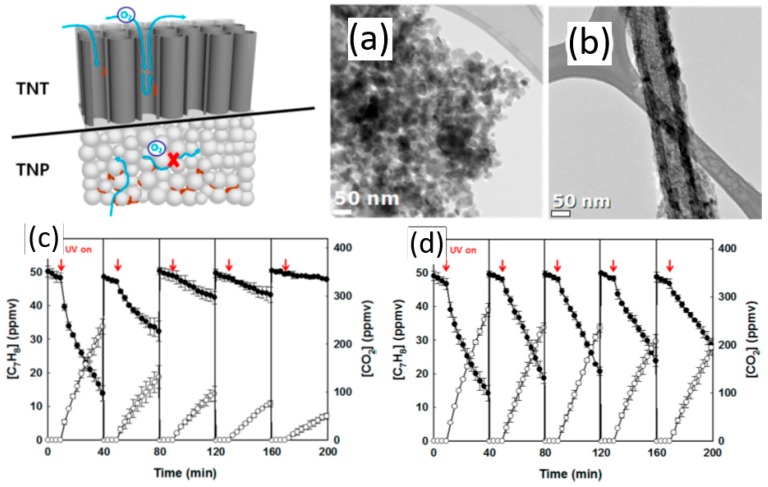
TEM images of fresh (**a**) TNP and (**b**) TNT; repeated photocatalytic degradation cycles of gaseous toluene on (**c**) TNP and (**d**) TNT in the air (●: [Toluene], ○: [CO_2_]) [36]. (adapted from [36], with permission from American Chemical Society, 2019).

TiO_2_ with different morphologies showed different photocatalytic performances. Lee et al. [35] developed novel nanostructured gas filtering systems with TiO_2_ thin films using atomic layer deposition (ALD) for VOCs, which showed a superior efficiency for the toluene adsorption. Weon and Choi [36] compared the photocatalytic activities of TiO_2_ nanotubes (TNT) and TiO_2_ nanoparticles (TNP) film during the repeated cycles of photocatalytic degradation of gaseous toluene and acetaldehyde. Figure 3a,b shows the TEM images of fresh TNP and TNT, respectively. The photocatalytic activity of TNT showed only moderate reduction after the five cycles of toluene degradation, whereas TNP underwent rapid deactivation as the photocatalysis cycles were repeated, even in a more oxidizing atmosphere (Figure 3c,d). With a highly-ordered open channel structure, TNT can easily supply O_2_ molecules to the active sites with less mass transfer limitation, which prevents the TNT surface from carbonaceous residues accumulation. It indicated that the structural characteristics of TNT are highly advantageous in preventing the catalyst deactivation during the photocatalytic degradation of aromatic compounds. Weon et al. [37] later synthesized freestanding doubly open-ended TiO_2_ nanotubes (DNT) film, which exhibited higher activity and durability for the photocatalytic degradation of gaseous acetaldehyde and toluene than TiO_2_ nanotubes. If the freestanding DNT film was additionally loaded with TiO_2_ nanoparticles (NP@DNT) in the inner wall, the activity for VOC degradation will be increased by 1.3 and 1.8 times of those for bare DNT and bare TNT, respectively. However, the loading of TiO_2_ nanoparticles on TiO_2_ nanotubes showed a lower activity than bare TNT.

TiO_2_ primarily exists in three crystal phases: anatase, brookite and rutile. Among them, the anatase form appears to be the most photoactive and the most practical for widespread environmental applications [25]; the brookite was once regarded to not be suitable as a photocatalyst [25,61], and later the successful synthesis of nanostructured brookite was showed to greatly enhance the photocatalytic performance [62]. Wu et al. [38] investigated the synergetic effect between anatase and rutile nanoparticles in gas-phase photocatalytic oxidations of hexane and methanol. The synergetic effect could be more significant if anatase and rutile particles are closely contacted. The long-term experiment proves the stability of the photocatalyst activity, and it cannot be improved by sulfation, which works well for the single-phase anatase TiO_2_.

**Figure 4:**
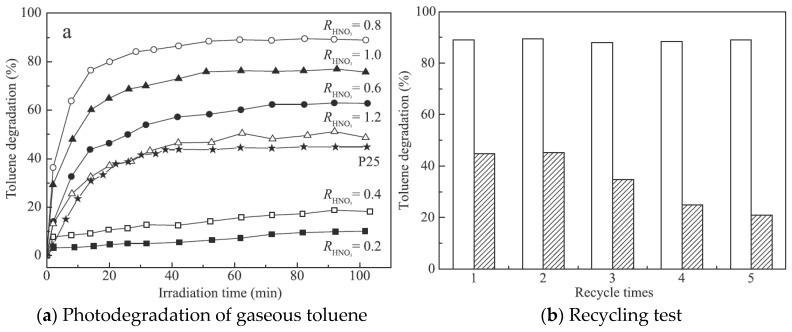
Comparison results between TiO_2_ and P25 for (**a**) photodegradation rate of gaseous toluene and (**b**) recycling test over tricrystalline TiO_2_-0.8 (blank) and P25 (filled) for five repeat uses [39]. (adapted from [39], with permission from Elsevier, 2019).

The tricrystalline TiO_2_ shows higher photocatalytic activity and durability toward gaseous toluene than bicrystalline TiO_2_ [39]. To remove toluene from the indoor air efficiently and economically, Chen et al. [39] synthesized anatase/brookite/rutile tricrystalline TiO_2_ by a low-temperature hydrothermal route with HNO_3_. As shown in Figure 4a, the one with RHNO_3_ = 0.8 (80.7% anatase, 15.6% brookite and 3.7% rutile) had the highest photocatalytic activity about 3.85-fold higher than that of P25 TiO_2_, which is a widely-used benchmark model photocatalyst coexisting anatase and rutile phases. Moreover, the high activity did not significantly degrade even after five reuse cycles (Figure 4b).

**Figure 5:**
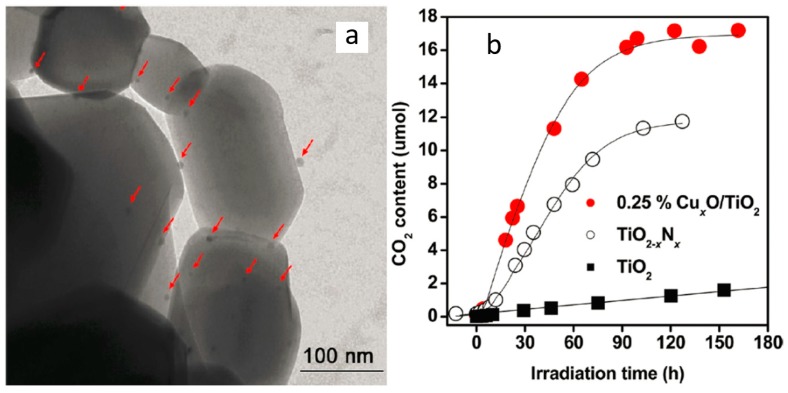
(**a**) TEM images of the 0.25% Cu_x_O/TiO_2_ sample. Cu_x_O clusters (marked by red arrows) were highly dispersed on the TiO_2_ surfaces; (**b**) comparative studies of CO_2_ generation over bare TiO_2_, TiO_2−x_N_x_, and 0.25% Cu_x_O/TiO_2_ samples under the same conditions [41]. (adapted from [41], with permission from American Chemical Society, 2019).

However, the leading semiconductor photocatalyst, TiO_2_, also suffers from low photocatalytic activity under visible light activation because of its intrinsic wide band gap. Therefore, to increase the efficiency of TiO_2_ in the visible light region, TiO_2_ is modified with various nanomaterials including other metal oxides [40,41,42], carbonaceous nanomaterials [43] etc. Zhong et al. [40] developed a TiNbON compound (band energy of 2.3 eV) using urea-glass synthesis as a visible light responsive photocatalytic oxidation material for toluene degradation. Experimental results showed that the visible light-driven catalyst was able to remove up to 58% of the toluene and generated less formaldehyde than the commercial TiO_2_ with reasonable durability and stability. Qiu et al. [41] grafted nano-Cu_x_O clusters onto TiO_2_ to generate an excellent risk-reduction material in indoor environments (Figure 5a). The Cu^II^ in the Cu_x_O clusters enables TiO_2_ to protoxidize VOCs under visible light efficiently, and it has the antimicrobial properties under dark conditions due to Cu^I^ species. Therefore, the efficient reduction of VOCs and antipathogenic activity could be achieved in hybrid Cu_x_O/TiO_2_ nanocomposites with a proper proportion of Cu^I^ and Cu^II^ in Cu_x_O. As shown in Figure 5b, the Cu_x_O/TiO_2_ sample shows a superior photocatalytic activity over the TiO_2−x_N_x_ sample with high quantum efficiencies and stability under long-term light irradiation.

**Figure 6:**
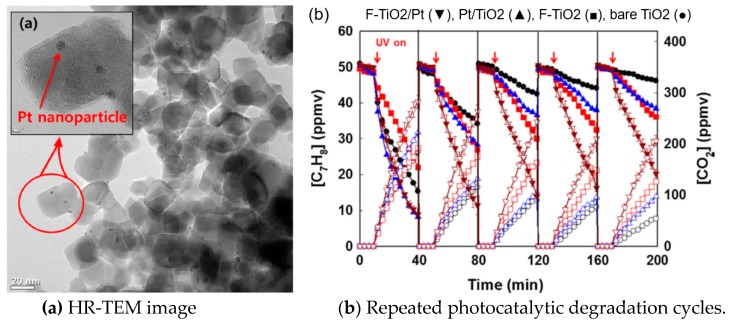
(**a**) HR-TEM image and (**b**) repeated photocatalytic degradation cycles of gaseous toluene on F-TiO_2_/Pt [42]. (adapted from [42], with permission from Elsevier, 2019).

Jo et al. [63] applied an annular reactor coated with unmodified or nitrogen (N)-doped TiO_2_ for VOCs removal in the indoor environment. The photocatalytic technique using N-doped TiO_2_ was much superior to that for unmodified TiO_2_, and it can remove above 90% for four target compounds (ethyl benzene, o,m,p-xylenes) under conditions of less humidified environments, including a typical indoor comfort range (50–60%). Weon et al. [42] modified TiO_2_ nanoparticles with Pt and fluoride and tested their durability for toluene removal. Figure 6a shows the HR-TEM of the composite. Although Pt/TiO_2_ had a higher photocatalytic degradation activity than bare TiO_2_, it could be deactivated rapidly during repeated cycles. Among them, F-TiO_2_/Pt showed the highest photocatalytic activity and durability for toluene degradation (Figure 6b).

**Figure 7:**
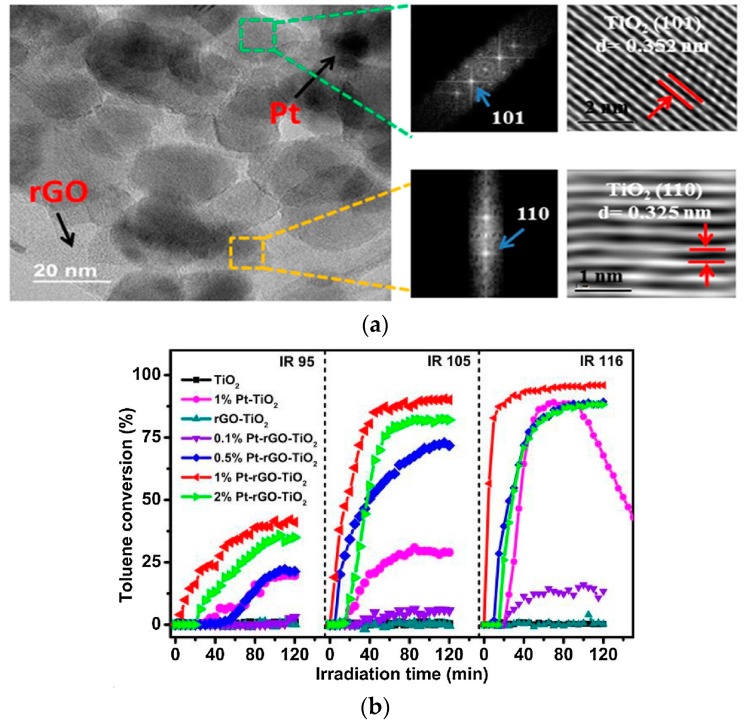
(**a**) High-angle annular dark-field scanning transmission electron microscopy images and HRTEM of 1% Pt-rGO-TiO_2_; (**b**) time course of toluene conversion over TiO_2_, 1% Pt-TiO_2_ and x% Pt-rGO-TiO_2_ (x = 0, 0.1, 0.5, 1 and 2) under IR irradiation with various light intensities (95, 106 and 116 mW/cm^2^) [43]. (adapted from [43], with permission from Elsevier, 2019).

Li et al. [43] introduced a hybrid nanomaterial Pt-rGO-TiO_2_ (Figure 7a). With a broad light wavelength absorption (800–2500 nm), the highly-active photo-thermal responsive catalyst can decompose VOCs efficiently under IR irradiation. As shown in Figure 7b, the light intensity can affect the efficiency of Pt-rGO-TiO_2_ composites on the conversion of toluene and the yield of CO_2_. If the infrared irradiation intensity is 116 mW/cm^2^, a maximum photo-thermal conversion efficiency of 14.1% would be achieved with a significant toluene conversion of 95% and a CO_2_ yield of 72%, and a nearly 50 h stability duration.

**Figure 8:**
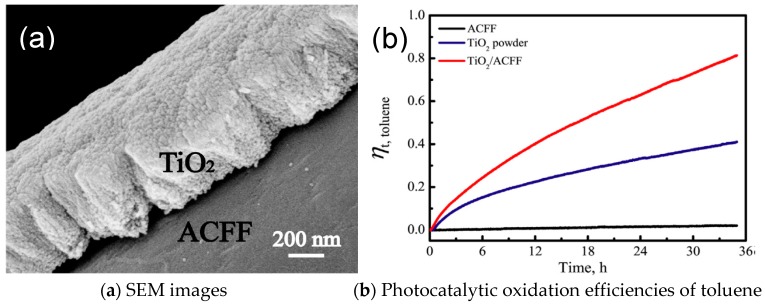
(**a**) SEM images and (**b**) Photocatalytic oxidation efficiencies of toluene as function of photocatalytic time under UV irradiation with TiO_2_/ACFF porous composites [6]. (adapted from [6], with permission from Elsevier, 2019).

Metal oxides also synthesized with adsorption materials to enhance their photocatalysis performances. Li et al. [6] firstly fabricated nanostructured TiO_2_/activated carbon fiber-felt (TiO_2_/ACFF) porous composites by the in-situ deposition of TiO_2_ microspheres on the carbon fibers in ACFF. Figure 8a shows hierarchical nanostructures constructed by nanocrystals of TiO_2_ microspheres. Due to the synergetic effects of nanostructured TiO_2_ and ACFF, the TiO_2_/ACFF porous composites possess excellent adsorption and photodegradation properties for toluene. The ACFF in the TiO_2_/ACFF porous composites significantly enhances photocatalytic property for toluene by hindering the recombination of electron-hole pairs, reducing the TiO_2_ band gap energy to 2.95 eV and accelerating toluene adsorption.

Similar combination can be found in Ref. [44], where the TiO_2_ nanoparticles (Ti-NP) were synthesized with decahedral anatase particles (DAPs), and their photocatalytic activity of TiO_2_/zeolite hybrids for VOCs oxidation was analyzed. TiO_2_ nanoparticles of 5 nm, DAPs of ca. 100 nm, and 1.0 μm clusters of TiO_2_ made of 15 nm mean particle size characterized the three types of TiO_2_. The incorporation of TiO_2_-NP into the zeolitic material led to composites with around 10 times more photoactivity that the single titania particles. Elfalleh et al. [64] used TiO_2_-impregnated polyester and glass fiber to address the photocatalytic degradation aldehydes (air-solid interface). Also, the TiO_2_ nanoparticles were fixed by the glass fiber tissue coated with colloidal silica in reactors to photo-catalytically remove isovaleraldehyde [65] and isovaleric acid [66].

#### 2.1.2. Zinc Oxide

**Figure 9:**
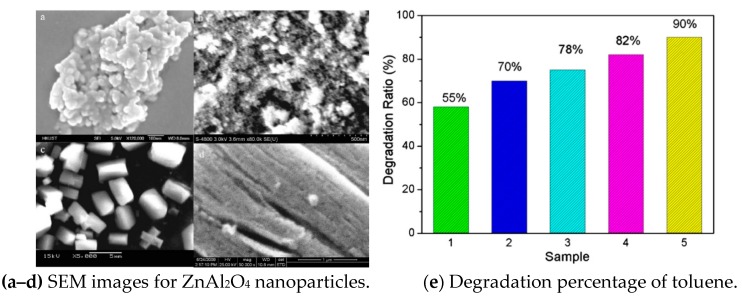
SEM images for ZnAl_2_O_4_ nanoparticles synthesized with (**a**) hydrothermal, (**b**) citrate precursors, (**c**,**d**) solvothermal synthetic methods. (**e**) The degradation percentage of toluene among 1 (ZnAl_2_O_4_ nanoparticles + citrate precursors), 2 (P25 TiO_2_), 3 (ZnAl_2_O_4_ nanoparticles + hydrothermal), 4 (TiO_2_ nanoballs), and 5 (ZnAl_2_O_4_ nanoparticles + solvothermal synthetic) samples under UV illumination [45]. (adapted from [45], with permission from Elsevier, 2019).

As an alternative to TiO_2_, zinc oxide is considered to be a fast and efficient chemical decontamination of VOCs [67]. Li et al. [45] compared three synthetic methods to prepare ZnAl_2_O_4_ for the photocatalytic degradation of gaseous toluene: solvothermal, citrate precursor and hydrothermal methods, whose SEM figures can be found in Figure 9a–d. The photocatalytic performances of the ZnAl_2_O_4_ samples synthesized by facile solvothermal method exhibited about 90% photocatalytic efficiency of toluene (Figure 9e). The photocatalytic oxidation of gaseous pollutant over UV-illuminated ZnAl_2_O_4_ is a promising technique for air purification.

#### 2.1.3. Nickel Oxide

**Figure 10:**
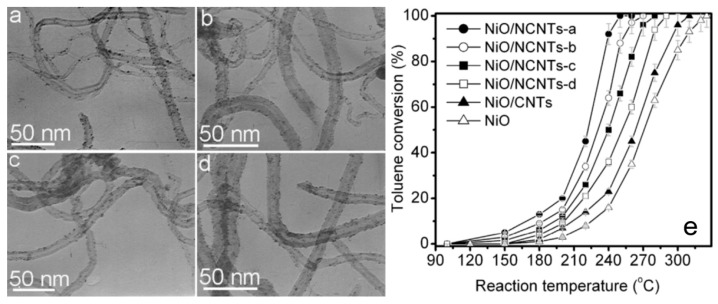
TEM images of NiO/NCNT catalysts with the pyridine to 3-(aminomethyl) pyridine volume ratios of (**a**) 5, (**b**) 3, (**c**) 1 and (**d**) 0; and (**e**) their toluene conversion vs. reaction temperatures against those of NiO/CNTs [46]. (adapted from [46], with permission from Elsevier, 2019).

Jiang et al. [46] compared the catalysis performance of nitrogen-doped carbon nanotubes (NCNTs) supported by NiO (NiO/NCNTs) with different pyridine volume ratios (Figure 10a–d). The oxygen adspecies concentration and low-temperature reducibility of NiO/NCNTs increased with increasing the doped graphitic-like N(NG) content of NCNTs (Figure 10e). The optimized NiO/NCNTs-d catalyst with NG content of 6.22 at.% can achieve a completed conversion of toluene at 248 °C, and has a TOF value of nearly 10 times NiO/CNTs at 160 °C.

#### 2.1.4. Tungsten Triocide

Kim et al. [47] combined nanodiamond (ND) with WO_3_ (as an alternative co-catalyst for Pt) to degrade VOCs under visible light. NDs-loaded WO3 showed a highly enhanced photocatalytic activity for the degradation of acetaldehyde (~17 times higher than bare WO_3_), which is more efficient than other well-known co-catalysts (Ag, Pd, Au, and CuO) loaded onto WO_3_ and comparable to Pt-loaded WO_3_. the surface conductivity of ND loaded on WO_3_ plays a critical role in the overall photocatalysis. The photocatalytic activity of NDs/WO_3_ was higher than that of WO_3_ loaded with other carbon-based co-catalysts (graphene oxide or reduced graphene oxide).

#### 2.1.5. Manganese Oxide

Genuino et al. [48] synthesized cryptomelane-type octahedral molecular sieve (OMS-2) manganese oxide, amorphous manganese oxide (AMO), and mixed copper manganese oxide (CuO/Mn_2_O_3_) nanomaterials, together with commercial MnO_2_. Due to complex reasons including structure, morphology, hydrophobicity, and redox properties, OMS-2, AMO, and CuO/Mn_2_O_3_ showed higher oxidative activities than the commercial MnO_2_. Miyawaki et al. [49] developed a novel hybrid catalyst for long-lifetime formaldehyde removal, which deposits manganese oxide (MnO_x_) catalysts on a polyacrylonitril-based activated carbon nanofiber (PAN-ACNF) support. The combination of MnO_x_ with PAN-ACNF induced synergic effects on the formaldehyde removal performance, which doubly improved the performance of PAN-ACNF in either dry or humid conditions without UV light. The manganese oxides were also interacted with cerium oxide [68] and CoAl mixed oxides [69] for a better catalytic removal of gaseous VOCs.

#### 2.1.6. Bi-Based Compounds

Qian et al. [50] incorporated highly stable carbon quantum dots (CQDs) with Bi_2_WO_6_ to sufficiently photocatalytic removal of VOCs. The CQDs/Bi_2_WO_6_ photocatalyst can extend the absorption into visible light region and improve the photoexcited charge separation in comparison with pristine Bi_2_WO_6_. This photocatalyst showed higher photocatalytic oxidation activities towards acetone and toluene under both UV–vis and visible light irradiation.

#### 2.1.7. Ag-Based Compounds

Kobayashi et al. [70] investigated the photocatalytic activity of AgBr. AgBr(N_2_) prepared at 250 °C showed the highest H_2_ generation activity, although larger crystallites of Ag were observed. Cao et al. [51] synthesized a novel AgBr/WO_3_ composite photocatalyst by loading AgBr on WO_3_ substrate via the deposition–precipitation method. AgBr/WO_3_ displays good photocatalytic activity under visible light (*λ* > 420 nm).

#### 2.1.8. Platinum Suported Material

**Figure 11:**
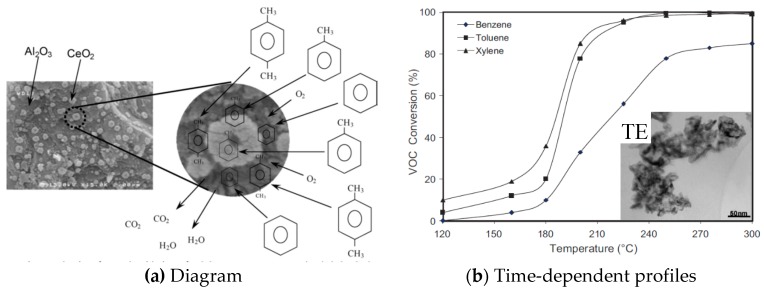
(**a**) Reaction mechanism for total oxidation of VOCs over nanostructured Pt/Al_2_O_3_–CeO_2_ catalysts. (**b**) Comparison of catalytic performance of synthesized Pt (1 wt%)/Al_2_O_3_–CeO_2_ (30 wt%) nanocatalyst for total oxidation of benzene, toluene and xylene [52]. (adapted from [52], with permission from Elsevier, 2019).

Abbasi et al. [52] prepared Pt/Al_2_O_3_–CeO_2_ nanocatalysts with Pt loading of 1% and ceria loading of 10, 20 and 30% to be utilized in catalytic oxidation of BTX (Figure 11a). The results of toluene oxidation indicated that the synthesized nanocatalysts were highly active and able to remove nearly 100% of toluene and xylene and about 85% of benzene as representative VOCs (Figure 11b).

Chen et al. [53] prepared a series of Pt/Al_2_O_3_ catalysts by modified ethylene glycol (EG) reduction approach with Pt particle sizes between 1.2–2.2 nm. Pt/Al_2_O_3_ catalyst with 1.2 nm Pt size exhibited optimum catalytic oxidation activity of benzene at 145 °C. The catalysts showed excellent adaptability for different VOCs and durability for benzene oxidation during the long-term continuous test, whether in dry air or in the coexistence of CO_2_, cyclohexane or H_2_O. Wang et al. [54] prepared a Pt/TiO_2_/Al_2_O_3_ catalyst on an anodic alumite plate for the catalytic decomposition of formaldehyde at ambient temperature. The developed catalyst has good activity on decomposing HCHO at ambient temperature. With quick activation of absorbing oxygen, the Pt/TiO_2_/Al_2_O_3_ catalyst showed a high activity towards the catalytic decomposition of formaldehyde at ambient temperature.

#### 2.1.9. Iridium Particles

Other oxides were also investigated, for example, Schick et al. [55] synthesized iridium particles supported on silica for the total oxidation of VOCs, and the catalytic activity increases when the iridium oxide particle size decreases.

### 2.2. Carbon-Based Materials

#### 2.2.1. Carbon-Based

**Figure 12:**
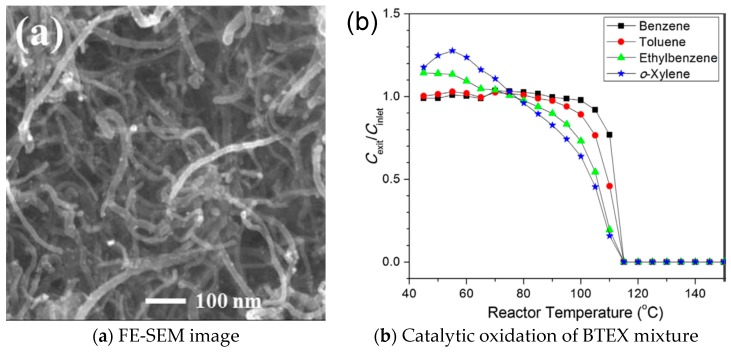
(**a**) Field emission scanning electron microscopy image and (**b**) catalytic oxidation of BTEX mixture as a function of reactor temperature over 30 wt% Pt/CNT catalyst [56]. (adapted from [56], with permission from Elsevier, 2019).

Joung et al. [56] fabricated a novel Pt/carbon nanotube (CNT) catalyst using a molecular-level mixing method (Figure 12a), and its performance on oxidation of benzene, toluene, ethylbenzene, and o-xylene (BTEX) was investigated at temperatures ranging from 40 to 150 °C (Figure 12b). The oxidation activity was presumably promoted because of the higher surface BTEX concentration afforded by the adsorption capability of multiwalled carbon nanotubes. The catalyst was characterized by its unique hydrophobic property, which facilitates the conversion of BTEX with high activity at relatively low temperatures and was unaffected by moisture in the system.

#### 2.2.2. Graphene and Graphene Oxide (GO)

Graphene and graphene oxide (GO) have been considered as a proficient matrix for sorption gaseous pollutants due to their advanced properties including facile synthesis method, high surface area, robust pore structure, lightweight, high chemical stability, and high thermal stability [71]. In recent years, great effort has been put into combining graphene/GO with various metal oxides (including TiO_2_, WO_3_, SnO_2_ and CO_3_O_4_ etc.) to improve the efficiency of VOCs’ removal. Even with unique structural and electronic properties, the metal oxide (e.g., TiO_2_/graphene) was found to be the same in essence as other TiO_2_/carbon (carbon nanotubes, fullerenes, and activated carbon) composite materials with regards to the enhancement of photocatalytic activity of TiO_2_ [57]. Since GO can be readily prepared from low-priced graphite materials on a huge scale, the usage of GO-based hybrid multifunctional materials should be much more profitable than that of other expensive nanomaterials such as functionalized carbon nanotubes [71].

Zhang et al. [57] prepared the nanocomposites of TiO_2_/graphene via a facile hydrothermal reaction of graphene oxide and TiO_2_ in an ethanol water solvent. This nanocomposite showed much higher photocatalytic activity and stability than bare TiO_2_ toward the gas-phase degradation of benzene, and the higher weight ratio in TiO_2_/graphene will decrease the photocatalytic activity. The photocatalytic efficiency can be strongly affected by the structure of graphene-based composites. Roso et al. [58] compared the performances of three graphene-based co-catalysts (graphene oxide, reduced graphene oxide, and few-layer graphene) on methanol gas-phase photooxidation. Among them, the reduced graphene oxide gave the best performance on degrading methanol with a higher rate.

## 3. Applications on Buildings

Photocatalysts can be applied on construction materials; their superior photoactivated properties can efficiently reduce or abate the harmful VOCs under UV light irradiation. These materials allowed both to degrade polluting compounds at the materials surface and to decrease maintenance costs thanks to their self-cleaning properties. As a main function for the self-cleaning applications, these materials such as TiO_2_ were coated on the surface or mixed with the building materials including glass, mortars, stone [72], asphalt and concrete [73,74], etc. Table 2 summarized the common applications of photocatalytic materials for VOC removal on buildings.

### 3.1. Indoor Air Treatment

Indoor air treatment can be used to filter the VOCs from the air to maintain a good quality of air. The filter can be coated with nanomaterials to achieve a better performance for VOCs oxidation. Li et al. [75] prepared a novel Pt/ZnO/SiC filter for the oxidation of toluene; the ZnO coating can disperse Pt nanoparticles and significantly enhance the photocatalytic performance. This filter can achieve complete conversion of toluene at a filtration velocity of 0.72 m/min within 240 h at 210 °C. TiO_2_ is more commonly used for application. Zadi et al. [76] evaluated the efficiency of non-thermal plasma and heterogeneous photocatalysis processes for indoor air treatment with glass fiber tissue-supported TiO_2_. Various impact factors were analyzed and the combination of plasma DBD and photocatalysis is proved to enhance the removal efficiency. Li et al. [77] found that the photocatalytic efficiency of the TiO_2_ catalysts with the lanthanide ion doping was remarkably enhanced by BTEX removal. By comparing different types of Ln^3+^–TiO_2_ (La^3+^/Nd^3+^–TiO_2_) with various lanthanide ion dosage, the 0.7% Ln^3+^–TiO_2_ catalysts showed the highest adsorption ability, and 1.2% Ln^3+^–TiO_2_ catalysts achieved the highest photocatalytic activity. Boyjoo et al. [78] also reviewed the catalyst used for air purification in 2017 and concluded that the most commonly used materials were TiO_2_ and ZnO, and only very few studies were using other photocatalysts.

### 3.2. Coating

Photocatalysts have multi-function when coated on the construction materials. For example, the TiO_2_ coatings on the glass surface can not only degrade organic dirt and VOCs under light, but also improve the surface hydrophilicity, which can efficiently remove the dust and degraded organic dirt by rainfall [79]. Oladipo et al. [80] reported the photocatalytic activity and energy efficiency of two self-cleaning glasses, Pilkington Activ™ Blue and Pilkington Activ™ Clear. The Clear one was more active than the Blue one towards 2-propanol oxidation under ultraviolet irradiation and showed the best reactivity in the degradation of pre-adsorbed stain under simulated solar irradiation. TiO_2_-coated exterior paints can contribute to a 90~98% decane degradation [81].

TiO_2_ can be coated to materials in air filters to enhance the photocatalytic oxidation [82]. Zhong et al. [83] compared the performances of two commercially available photocatalytic oxidation air filters, titanium dioxide (TiO_2_) coated on fiberglass fibers (TiO_2_/FGFs) and TiO_2_ coated on carbon cloth fibers (TiO_2_/CCFs) in a pilot duct system. The single-pass removal efficiency of these air filters ranks alcohols > ketones > aromatics > alkanes, and various influential factors were analyzed. Jiang et al. [84] coated the ceramic tiles with N, F and Fe ions-doped TiO_2_ to photo-catalytically remove NO under visible light. Both air pollutions of inorganic NO and organic compounds can be purified by the ceramic tiles. The coating enables ceramic tiles to have enhanced photocatalytic efficiency, low water adsorption performance, good fastness, and antibacterial capability to reduce the risk of bacterial infection.

To reserve the original aesthetic features of historical and monumental architectures, Munafo et al. [72] applied anatase TiO_2_ colloidal suspensions via spray-coating to deposit transparent self-cleaning coatings on stones. Photocatalytic performances of TiO_2_-based coatings with one and three spray cycles were compared against that of the untreated travertine. The self-cleaning ability of analyzed coatings was degraded by the ageing processes till reaching low efficiency, and it shows no significant difference between single-layer and multilayer coatings in long-term use. Results seem to encourage the use of nano-structured TiO_2_ for preserving stone during time, while the undercoat and new and more stable colloidal TiO_2_ is needed.

To achieve a high photocatalytic efficiency and a robust weathering resistant ability, Guo et al. [85] applied a transparent photocatalytic coating containing TiO_2_ particles on the architectural mortar; its photocatalytic performances was better than the TiO_2_ intermixed samples on air purifying and self-cleaning properties under both UV-A and visible light irradiation conditions, and their abilities showed no obvious deterioration during a simulated facade-weathering process.

### 3.3. Paints

The commercial TiO_2_, P25 (75% anatase and 25% rutile phases), is commonly applied in paints. Guo et al. [86] directly applied a TiO_2_-containing paint (clear in colour) on the surface of self-compacting architectural mortars (SCAM). The results showed that the TiO_2_ paint -oated SCAM sample displayed both a high photocatalytic rhodamine b removal ability and a robust weathering resistance under all conditions. Baudys et al. [87] investigated the photocatalytic properties of self-cleaning acrylic paint containing TiO_2_ and ZnO using Acid Orange 7 as a model compound. The photocatalytic activity of TiO_2_ increases with the weathering time. However, even if the initial photoactivity of the unweathered paints with ZnO was significantly higher, the photocatalytic activity decreased after weathering, due to the loss and/or photocorrosion of ZnO particles during the weathering process. Amorim et al. [88] synthesized an efficient and durable self-cleaning acrylic paint containing mesoporous TiO_2_ (MTiO_2_). The paint with MTiO_2_ incorporated showed a better photoactivity and higher durability than the reference paint with P25 added in the cyclic analysis, which indicated that MTiO_2_ microspheres can provide acrylic paint films with self-cleaning properties without severe degradation of the binder.

The self-cleaning photocatalytic paints could be designed for specific applications for outdoor or indoor environments. Most of these photocatalytic paints are based on titanium dioxide, however, the large band gap of TiO_2_ requires UV photons for the electrons-holes generation, which makes it difficult to be efficient in the indoor environment. Galenda et al. [89] firstly compared the photocatalytic activity tests of indoor commercial self-cleaning paints under actual indoor light. These paints contain titanium dioxide with different amounts and crystallographic forms. The results of experiments with different lights suggest that all samples are scarcely active under visible light and the pollutant probes are selectively bleached due to their sensitizing effect. Consequently, the pollutants’ ability in injecting electrons in the TiO_2_ conduction band deeply affects their removal.

Paolini [90] propose a pre-treatment with nitric or sulfuric acid of commercial TiO_2_ nanopowders to prevent from aging used in coating, mortars, or paints. photocatalytic performances for experiments with various diffuse reflectance of nitric and sulfuric acid were compared. Nitric acid causes a decrease in crystallinity and photocatalytic activity, which drops by almost 20%; and sulfuric acid is the best candidate for TiO_2_ nanoparticles acid treatment with the aim of improving both their reflectance in a wavelength region unaffected by aging and sustaining their photocatalytic activity over time.

### 3.4. Construction Materials

Diamanti et al. [91] investigated the photocatalytic and self-cleaning activity of colored mortars containing TiO_2_, and its possible interaction with iron oxide pigments, which are commonly added to the mixture in the production of colored mortars. Because of the self-cleaning characteristics, TiO_2_ containing mortars have lower soiling in atmospheric exposure. However, the Iron oxide pigments caused lower photocatalytic activity compared to white mortars. Nath et al. [92] reviewed the photocatalysts used in concrete and found that majority of the work in concrete adopted TiO_2_ as the photocatalyst. They also regarded the semiconductor oxide LiNbO_3_, which has been used in electronic instruments to replace TiO_2_ for artificial photosynthesis, as a promising and relatively new approach to be used in future concrete.

For the applications of photocatalysts on buildings, it is clear that the photocatalyst TiO_2_ dominates the application market, and very few applications adopt other catalysts such as ZnO. Due to its low cost, high chemical stability and non-toxic properties, TiO_2_ has been investigated extensively and first applied to the practical applications on buildings. Since various materials have different properties, other materials will also be applied in the near future according to their characteristics.

## 4. Conclusions

Thermal catalytic and photocatalytic nanomaterials are increasingly being used as an efficient approach to remove the VOCs, and their emerging applications on building and construction materials are promising for their air-cleaning properties. Among these catalysts, TiO_2_ is the most common, economical and efficient one due to its low cost, high chemical stability, and non-toxic properties. TiO_2_ has been investigated for decades and dominates the applications of catalytic VOC removal on buildings. Since other thermal catalysts also showed brilliant performance and have been studied continuously, there is expected to be more applications on buildings using other emerging catalysts rather than TiO_2_. As the photocatalysis performances of metal oxides can be enhanced by the synergy with hybrid adsorption materials [6], more catalysts combining different nanomaterials are expected. Among various applications of catalytic nanomaterials on buildings, the approach of coatings is more efficient and prevalent compared to intermixing [85] on the removal of VOCs. In order to remove the indoor VOCs more efficiently, more researches and applications should be done to increase their efficiencies in specific conditions such as visible light.

## Figures and Tables

**Table 1:** Commonly used materials for catalytic removal of VOCs.

No.	Catalytic	Category	VOC	Nanomaterial	Morphology	Medium	Doping Concentration	Synthesis	Ref.
1	Photo-	TiO_2_	Trichloro-ethylene	nanostructured TiO_2_ particles	Primary particle size: 2.3–30 nm, secondary particle size: 100–900 nm	titanium isopropoxide	water concentrations: 2.3, 0.3, 0.27, and 0.18 M	low-temperature synthesis, modified sol–gel method	[33]
2	Photo-	TiO_2_	Toluene	Titanium isopropoxide	Primary particle size:11 nm	isopropanol–water solution	2.5 mL H_2_O, 25 mL ethanol, 150-mL (hydrothermal)	sol–gel synthesis, thermal & hydrothermal methods	[34]
3	Photo-	TiO_2_	Toluene	TiO_2_ thin films	particle sizes less than 100 nm, monocrystalline nanodiamond	Titanium (IV) tetraisopropoxide (TTIP) (Ti(OCH(CH_3_)_2_)_4_) and water		detonation method (purchased from microdiamant)	[35]
4	Photo-	TiO_2_	Toluene, acetaldehyde	TiO_2_ nanotubes (TNT) & nanopartcles (TNP) film; commercial TiO_2_ (P25)	average surface area of 50 m^2^ g^−1^, primary particle size: 20–30 nm, channel pores diameter: 40–60 nm, tube length: 9.5 (±0.9) μm.	[TNP] Ethanol [TNT] ethylene glycol electrolyte	[TNP] 0.15 g/mL [TNT] 1st anodization: 0.5 wt% NH_4_F and 3 wt% H_2_O; 2nd: 0.3 wt% NH_4_F and 1 wt% H_2_O.	[TNP] doctor-blade method [TNT]two-step electrochemical anodization	[36]
5	Photo-	TiO_2_	Toluene	Ti-foil (99.7%,0.25 mm, Aldrich, USA)	top and bottom opened structure of which thediameters are 100 nm and 50 nm, respectively NP@DNT films of 15 (±2) μm	ethylene glycol solution containing 0.25 wt% NH4F and 0.3 vol% distilled water		potentiostatic anodization method	[37]
6	Photo-	TiO_2_	Hexane, methanol	anatase and rutile TiO_2_ (0.1 mol)	Surface area between 39 to 84 m^2^/g (given in table)	1.5 mol anhydrous Ethanol, water–ethanol solution containing 1 mol ethanol with a ratio of water:butoxide = 50:1.	aqueous HNO_3_ solution of various concentration (0.1–1.0 mol/L) with the ratio of solid (g): liquid (mL) = 1:10	hydrothermal method	[38]
7	Photo-	TiO_2_	Toluene	Anatase/brookite/rutile tricrystalline TiO_2_	amorphous TiO_2_ suspension	HNO_3_ solution (65%)	The molar ratios of HNO_3_ to TBOT (RHNO3) were varied from 0.2 to 1.2 at intervals of 0.2 by varying the volume of HNO_3_ solution.	low-temperature hydrothermal method	[39]
8	Photo-	TiO_2_	Toluene	co-alloying TiO_2_	fine bright yellow powder, primary particles diameter: 1–2 μm	TiCl_4_ reacted with NbCl5 and urea in an ethanol solution	toluene concentrations: 1~5 ppm; relative humidity: 25~65%; air velocity: 0.78~7.84 cm/s; irradiancy: 42~95 W/m^2^.	urea-glass synthesis	[40]
9	Photo-	TiO_2_	Isopropyl alcohol	Hybrid Cu_x_O/TiO_2_ Nanocomposites	Commercial TiO_2_ (rutile phase, 15 nm grain size, 90 m^2^/g specific surface area)	CuCl_2_ solution, NaOH and glucose solutions (reduce & control the Cu^I^/Cu^II^ ratio	10 mL of CuCl_2_ solution. Weight fraction of Cu: TiO_2_ is 1 × 10^3^: 2 × 10^2^.	simple impregnation method	[41]
10	Photo-	TiO_2_	Toluene	commercial TiO_2_ (P25)	Platinum nanoparticles in the size of 1–3 nm were clearly deposited on the surface of TiO_2_	0.5 wt% Pt and 30 mM fluoride for VOC degradation	sodium fluoride (10, 30, and 50 mM) and Pt (0.1, 0.5, and 1 wt%)	photo deposition method	[42]
11	Photo-	TiO_2_	Toluene	hybrid nanomaterial Pt-rGO-TiO_2_	TiO_2_ nanopowder: commercial P25 (Degussa).	ethanol-water	0.1, 0.5, 1 and 2 wt% Pt-rGO-TiO_2_ nanocomposite catalysts	solvothermal method	[43]
12	Photo-	TiO_2_	Toluene	Composites ACFF 0.5 mL tetra-butyl titanate (97 wt%)	Diameter: 12 μm, pore size: 32 μm.	Polytetrafluoroethylene (Teflon)-lined stainless-steel autoclaves	1.0, 2.0, 3.5 and 5.0 l of toluene were injected into the above reactor	Purchased ACFF,	[6]
13	Photo-	TiO_2_	Formaldehyde, trichloro-ethylene	TiO_2_ nanoparticles	BET area:392 m^2^ g^−1^, micro mean pore size: 0.6 nm	8 wt% DAPs		incipient wetness impregnation, freeze-drying, or mechanical mixing	[44]
14	Photo-	Zinc oxide	Toluene	ZnAl_2_O_4_ nanoparticles	commercial P25 powder (reference) TiO_2_ nanoballs in anatase phase	[solvothermal synthetic] Al(NO_3_)_3_·9H_2_O (2 mmol), Zn(NO_3_)_2_·6H_2_O (1 mmol), ethylene glycol (30 mL) [citrate precursors] 0.01 M Zn(NO_3_)_2_·6H_2_O, 0.02 M Al(NO_3_)_3_·9H_2_O, 100 mL DI water [hydrothermal] an equimolar amount of Zn(NO3)_3_·6H_2_O (2 mmol), Al(NO_3_)_3_·9H_2_O (4 mmol), urea[CO(NH_2_)_2_] (20 mmol) and deionized water (80 mL)		solvothermal, citrate precursor, hydrothermal methods	[45]
15	Photo-	Ni oxide	Toluene	Nitrogen-doped carbon nanotubes (NCNTs) supported NiO(NiO/NCNTs)	NCNTs: tubular structure, 20 nm-diameter; NiO: crystallite, 3–10 nm	catalyst and pyridine and/or 3-(aminomethyl)pyridine	volume ratio of pyridine to 3-(aminomethyl)pyridine: 5, 3, 1 and 0	Chemical vapor deposition method	[46]
16	Photo-	WO_3_	H_2_O_2_	Nano-diamonds combined with WO_3_	ND: ca. 4–6 nm diameter	WO_3_ (Aldrich)	0.5–16 wt% ND contents	Simple dehydration condensation	[47]
17	Photo-	Manganese Oxide	Benzene, Toluene, Ethylbenzene, Xylenes	Manganese Oxide and Copper	KMnO_4_ solution (OMS-2); Mn(CH_3_COO)_2_ 4H_2_O (AMO)	Mn(CH_3_COO)_2_ solution (OMS-2); KMnO_4_ (AMO);		a simple refluxing method	[48]
18	Photo-	Manganese Oxide	Formaldehyde indoors	manganese oxide	Shown in SEM images	ethanol solution of manganese acetate tetrahydrate (Mn(CH_3_COO)_2_·4H_2_O	Mn(CH_3_COO)_2_·4H_2_O:PAN-ACNF 0.5–20 wt.%		[49]
19	Photo-	Bi-based compounds	Acetone, toluene	Bi_2_WO_6_	CQDs: high dispersion, uniform size of 3–5 nm in diameter	carbon quantum dots (CQDs)	adding 1.0–6.0 g of CQDs	Hydrothermal synthesis	[50]
20	Photo-	AgBr	methyl orange	AgBr	monoclinic WO_3_ substrate, face-centered cubic AgBr nanoparticles: crystalline sizes less than 56.8 nm.	WO_3_	AgBr contents were respectively obtained and defined as TA-0.05, TB-0.10, TC-0.15, TD-0.20, TE-0.25, TF-0.30 and TG-0.40.	deposition–precipitation method	[51]
21	Thermal	Platinum	Toluene	Pt/Al_2_O_3_–CeO_2_ nanocatalysts	average size: 5–20 nm.	CeO_2_(10%)/Al_2_O_3_, 2.8 g Ce(NO_3_)_3_·6H_2_O, 100 mL distilled water	ceria loading of 10, 20 and 30%	wet impregnation method	[52]
22	Thermal	Platinum	benzene	Pt/Al_2_O_3_	Pt particle sizes between 1.2–2.2 nm	H_2_PtCl_6_·6H_2_O	Pt/A l_2_O_3−x_, x: pH value of 7.0, 9.0 and 11.0	modified ethylene glycol (EG) reduction approach	[53]
23	Thermal	Platinum	Formaldehyde (HCHO)	Pt/TiO_2_/Al_2_O_3_	BET area from 16.5 to 182.5 m^2^/g	(NH_4_)[TiO(C_2_O_4_)_2_]	The platinum loading: 0.62, 1.26,1.19 and 1.25 gm^−^^2^	Electro-deposition technology	[54]
24	Thermal	Silica-iridium	Toluene	chloride-ion free iridium acetylacetonate, Ir(AcAc)_3_	∼5 to 27 nm	SiO_2_ Degussa Aerosil 200	Size of iridium particles: ~5 to 27 nm (calcination temperature 350~750 °C)	incipient wetness impregnation	[55]
25	Thermal	Carbon	benzene, toluene, ethylbenzene, and oxylene	Pt/carbon nanotube (CNT) Multiwalled carbon nanotubes (MWCNT)	CNTs: 20–50 nm column diameters MWCNTs: 20–50 nm diameters	acid treatment using HF, H_2_SO_4_, and HNO_3_	Pt content in the catalysts ranging from 10 to 30 wt%.	a molecular-level mixing method	[56]
26	Photo-	Carbon based	Volatile Aromatic Pollutant	TiO_2__graphene	Shown in SEM image	An ethanol-water solvent	P25_GR with weight addition ratios of 0.2, 0.5, 1, 2, 5, 10, and 30% GR.	facile hydrothermal reaction	[57]
27	Photo-	Carbon-based	methanol	graphene oxide, reduced graphene oxide, and few-layer graphene	BET area (m^2^/g): rGO+TiO_2_: 49.34, GO+TiO_2_: 43.79, G+TiO_2_: 41.54	Polyacrylonitrile	a polymer concentration of 5% (*w*/*w*) in *N*,*N*-dimethylformamide.	hydrothermal method (reduced graphene oxide); others purchased	[58]

**Table 2:** Applications of photocatalytic materials for VOC removal on buildings.

No.	Catalytic	Applications	Materials	Comparison & Experiments	Pollutants	Performance	Ref.
1	Thermal	Indoor air purification	Pt/ZnO/SiC	Toluene concentration: 100~500 ppm Loading of Pt nanoparticles:0.030 wt% (Pt/ZnO/SiC) ~0.017 wt% (Pt/SiC).	Toluene	Toluene was used as a model volatile organic compound and reached complete conversion of up to 100% over the porous tubular Pt/ZnO/SiC material at a filtration velocity of 0.72 m/min within 240 h at 210 °C maintained within 24 h	[75]
2	Photo-	Indoor air purification	Glass fiber tissue supported TiO_2_	Inlet pollutant concentrations (25–300 mg m^−3^), flow rates (2–8 m^3^ h^−1^), relative humidity of effluent (5, 30, 50 and 90%), input of the plasma discharge (9–21 kV)	Trichloromethane (CHCl_3_)	Combination of plasma DBD and photocatalysis enhances the removal efficiency	[76]
3	Photo-	Indoor air purification	Ln^3+^–TiO_2_	La^3+^–TiO_2_ and Nd^3+^–TiO_2_ Lanthanide ion dosage of 0.7%, 1.2%, 1.6% and 2.0%	benzene, toluene, ethylbenzene and o-xylene (BTEX)	Highest adsorption ability: 0.7% Ln^3+^–TiO_2_ catalysts. TiO_2_ photocatalytic efficiency with the lanthanide ion doping was remarkably enhanced by BTEX removal. The 1.2% Ln^3+^–TiO_2_ catalysts achieved the highest photocatalytic activity. Residence time: 72 s using 1.2% La^3+^–TiO_2_ catalyst	[77]
4	Photo-	Coating	TiO_2_ thin films	Commercial glasses: Pilkington Activ™ Blue PAB) and Pilkington Activ™ Clear (PAC).	2-propanol	For the 2-propanol oxidation, PAC was found to be more active under UV light due to the larger surface area and higher TiO_2_ particle concentration.	[67]
5	Photo-	Coating	TiO_2_ coated on fiberglass fibers	TiO_2_ coated on carbon cloth fibres, a pilot duct system for experiment	polytetrafluoroethylene	The single-pass removal efficiency ranks: alcohols > ketones > aromatics > alkanes.	[69]
6	Photo-	Coating	TiO_2_	single-layer coating & multilayer TiO_2_ coating	rhodamine B	Degrading self-cleaning ability of analysed coatings caused by ageing processes, and no significant difference between single-layer and multilayer coatings in the long-term	[63]
7	Photo-	Paint	Three self-cleaning photocatalytic paints	Three white commercial photocatalytic paints; expose to UVC lamp, Xenon lamp, LED and fluorescent lamps for 10 h	methyl red, methylene blue	Limited photocatalytic action under visible light	[74]
8	Photo-	Paint	commercial AEROXIDE_TiO_2_ P25 powder	Matrix with nitric acid and H_2_SO_4_	rhodamine b	Nitric acid causes a decrease in crystallinity and photocatalytic activity, which drops by almost 20%; H_2_SO_4_ the best candidate for TiO_2_ nanoparticles acid treatment	[75]
9	Photo-	Paint	TiO_2_ microspheres	commercial TiO_2_ particles P25	methylene blue solution	MTiO_2_: more stable and better photoactivity	[73]
10	Photo-	Paint	TiO_2_	5% P25-TiO_2_-intermixed and dip-coated SCAM samples	rhodamine b	TiO_2_/SCAM: high self-cleaning ability and a robust weathering resistance under UV-A and visible light irradiations.	[71]
11	Photo-	Paint	TiO_2_ coating (PC-S7, Cristal Active)	TiO_2_ (P25) intermixed nanopowder. Experiments: air purifying (indicated by NO_x_ removal) and self-cleaning (indicated by Rhodamine b removal).	Rhodamine b NO_x_	TiO_2_ coating on mortar shows better photocatalytic performances than TiO_2_ intermixed samples on air purifying and self-cleaning properties under both UV-A and visible light (VL) irradiation conditions.	[70]
12	Photo-	Paint	TiO_2_ P25	ZnO Experiments: Paints were exposed to simulated weathering tests in a QUV panel	dye Acid Orange 7	Photocatalytic activity of TiO_2_ increases with weathering time. ZnO: significantly higher photocatalytic activity for initial photoactivity of the unweathered paints but decreased after weathering.	[72]
13	Photo-	Mortar	Mortars containing TiO_2_ and iron oxide pigments	Atmospheric exposure tests and photocatalytic degradation tests were performed.	2-propanol	Iron oxide pigments caused lower photocatalytic activity compared to white mortars. TiO_2_ + mortars has lower soiling in atmospheric exposure	[76]
